# Increased Cord Blood Betatrophin Levels in the Offspring of Mothers with Gestational Diabetes

**DOI:** 10.1371/journal.pone.0155646

**Published:** 2016-05-19

**Authors:** Xuemei Xie, Hongjie Gao, Shimin Wu, Yue Zhao, Caiqi Du, Guandou Yuan, Qin Ning, Kenneth McCormick, Xiaoping Luo

**Affiliations:** 1 Department of Pediatrics, Tongji Hospital, Tongji Medical College, Huazhong University of Science and Technology, Wuhan, China; 2 Department of Endocrinology and Metabolism, Affiliated Hospital of Guilin Medical University, Guilin, Guangxi, PR China; 3 Department of Hepatobiliary Surgery, Guilin Medical University, Affiliated Hospital, Guilin, Guangxi, 541001, P.R. China; 4 Department of Infectious Diseases, Tongji Hospital, Tongji Medical College, Huazhong University of Science and Technology, Wuhan, China; 5 Department of Pediatrics, Division of Endocrinology, University of Alabama at Birmingham, Birmingham, Alabama, United States of America; Virgen Macarena University Hospital, School of Medicine, University of Seville, SPAIN

## Abstract

**Aim:**

Exposing a fetus to hyperglycemia can increase the risk for later-life metabolic disorders. Betatrophin has been proposed as a key regulator of pancreatic beta cell proliferation and lipid regulation. Highly responsive to nutritional signals, serum betatrophin concentrations have been found to be altered by various physiological and pathological conditions. We hypothesized that betatrophin levels are increased in the cord blood in offspring exposed to intrauterine hyperglycemia.

**Methods:**

This was a cross-sectional study including 54 mothers who underwent uncomplicated Cesarean delivery in a university hospital. Maternal gestational glucose concentration was determined at 24–48 weeks gestation after a 75-g OGTT. Cord blood and placental tissue was collected immediately post delivery. Metabolic parameters were determined in the Clinical Laboratory. Cord blood betatrophin levels were assayed using a commercially available ELISA kit. Placental mitochondrial content was determined by real-time PCR.

**Results:**

Cord blood betatrophin levels were increased in the gestational diabetes mellitus (GDM) group compared with the normoglycemic group. Furthermore, betatrophin levels were positively correlated with maternal gestational 2h post-OGTT glucose, cord blood insulin, HOMA-IR, and inversely correlated with placental mitochondrial content.

**Conclusions:**

Cord blood betatrophin may function as a potential biomarker of maternal intrauterine hyperglycemia and fetal insulin resistance, which may presage for long-term metabolic impact of GDM on offspring.

## Introduction

It have been well documented that early-life environment play a substantial role in adult health [1–3]. Gestational diabetes mellitus (GDM), a common pregnancy complication, affects <1%– 28% of all pregnancies, the incidence depending on the diagnostic criteria and ethnicity [4], and parallels globally the obesity pandemic [5]. GDM contribute to the long-term metabolic derangements both in mother and child [6–10]. Notably, perturbations in glucose and lipid metabolism may manifest early in children exposed to intrauterine hyperglycemia [11–14]. The mechanism underlying the long-term metabolic effect of GDM remains uncertain.

Betatrophin (aka ANGPTL8) may play a role in pancreatic beta cell proliferation and lipid regulation [15]. It is a circulating 198 amino acid protein, highly expressed in liver and adipose tissue [15, 16]. Robustly responsive to nutritional signals, betatrophin levels have been found to be altered by various physiological and pathological conditions. For example, in humans, betatrophin concentrations are increased in type 2 diabetes [17–19], obesity [17], and type 1 diabetes [20], as well as in the postprandial state [17].

Pancreatic beta cells proliferation—dictated by numerous cell cycle genes—can be induced by physiological challenges such as gestation [21] and hyperglycemia [22]. Insulin resistance is yet another inducer. Using a mouse insulin resistance model created by an insulin receptor antagonist, Yi et al. reported that betatrophin promotes a compensatory beta cell proliferation [23]. Notwithstanding this initial study, the notion that betatrophin can augment beta cell mass is now contestable: betatrophin knockout mice rendered insulin resistant can still undergo beta cell proliferation [24]. Hepatic betatrophin expression increases in parallel with the higher beta cell replication rates over the course of gestation in mice [23]. Conceivably, betatrophin concentrations are also increased in the cord blood from GDM mothers, perhaps serving as a harbinger of future metabolic dysregulation. Previous studies in mice also indicate that betatrophin may alter lipid metabolism, especially triglycerides (TG). Betatrophin knockout mice have decreased TG levels in the fed state, reduced very low density lipoprotein (VLDL) secretion, and elevated LPL activity [25]. Antithetically, betatrophin overexpression in mouse liver increased serum TG levels [26]. Type 2 diabetes [27] and GDM [28] are associated with dyslipidemia, and diabetes patients have altered circulating betatrophin levels. Thus, betatrophin may be involved in lipid dysregulation in type 2 diabetes as well as GDM.

Herein we investigated the alteration of cord blood betatrophin levels in offspring exposed to intrauterine hyperglycemia, and explored the correlation between betatrophin and various metabolic parameters including maternal gestational blood glucose, BMI, cord blood lipid profile and homeostasis model assessment of insulin resistance (HOMA-IR). We hypothesized that cord blood betatrophin levels would be increased in the offspring from GDM mothers, and significantly correlated with maternal gestational glucose levels as well as other abovementioned metabolic parameters.

## Patients and Methods

### Study design and Participants

Enrollment consisted of women with singleton pregnancy who underwent uncomplicated Cesarean delivery at Tongji Hospital in Wuhan, China, from August 2013 to October 2013. All mothers are from Chinese Han ethnicity and had undergone a 75-g OGTT following the standard protocol at 24–28 weeks of gestation. According to the criteria set by International Association of Diabetes and Pregnancy Study Groups (IADPSG) [29, 30], GDM was diagnosed when the fasting plasma glucose ≥5.1 mmol/L or 1h post-OGTT glycemia ≥10.0 mmol/L or 2h post-OGTT glycemia ≥8.5 mmol/L. Women were excluded from the study if < 20 or > 40 years of age, or had been previously diagnosed with any disorder known to affect glucose metabolism including diabetes, GDM, polycystic ovarian syndrome, uncontrolled thyroid or liver disease. Mothers with a history of smoking or any substance abuse during the current pregnancy, or if there was premature delivery (<37 wk gestation) or birth weight < 2500 g were likewise excluded. Furthermore, participants with pregnancies resulting from in vitro fertilization or exposure to dexamethasone during pregnancy were excluded for the potential effect on offspring glucose metabolism. Additionally, participants with unavailable maternal gestational glucose records or if a cord blood sample was not obtained were ineligible. Only women undergoing uncomplicated Cesarean deliveries were recruited to exclude the influence of different mode of delivery on cord blood glucose. In total, data from 54 enrollees was available for analysis. All mothers with GDM were treated solely with diet in this study.

Cord blood and placental tissue were collected immediately after delivery. Cord blood was collected from the umbilical vein following standard protocols. Thereafter, the cord blood was centrifuged at 4°C. Insulin and glucose were measured within 2 hours. A portion of plasma and serum were frozen and stored at -80°C immediately after the centrifugation. Placenta tissue from the fetal side were obtained as previously described [31]. The samples were rinsed in normal saline at 4°C to remove excess maternal blood. Thereafter, samples were frozen in liquid nitrogen and stored in freezer of -80°C.

Most of the patients in this report were from previously reported cohorts [31]. All study procedures were approved by the Ethic Committee of Tongji Hospital, Huazhong University of Science and Technology (Approval number: TJ-C20130711), and written informed consent was obtained from each participant in accordance with the Declaration of Helsinki as revised in 2008.

### Laboratory measurements

All parameters were determined in the Clinical Laboratory of Genetic Metabolic Disease (Tongji Hospital, Huazhong University of Science and Technology). Blood glucose was measured using a glucose-oxidase/peroxidase method (Biosino Bio-Technology and Science Inc, Beijing, China), and the inter-assay and intra-assay CV were 3% and 2%, respectively. Insulin concentrations were determined using a chemiluminescent immunoassay (Beckman Coulter Inc., Brea, CA), with the inter-assay and intra-assay CV 3.5–4.5% and 2–2.6%, respectively. The homeostasis model assessment of insulin resistance (HOMA-IR) was calculated as: HOMA-IR = fasting glucose (mmol/L) ×fasting insulin (mU/L)/22.5. Serum total cholesterol (TC) and triglyceride (TG) were assayed using an enzymatic colorimetric method (Roche Diagnostics, Penzberg, Germany).The inter-assay CVs were 1.7% (TC) and 1.9% (TG) and intra-assay CVs were 0.8% (TC) and 1.6% (TG). HDL-cholesterol (HDL-C) and LDL cholesterol (LDL-C) were determined using commercially available kits (SEKISUI MEDICAL CO., Tokyo, Japan), and the inter-assay and intra-assay CVs were ≤10% and ≤5%, respectively. Apolipoprotein A and B (Apo-A and Apo-B) were quantified by immunoturbidimetric methods (Roche Diagnostics, Penzberg, Germany). The intra-assay CV were 0.7–1.8% (Apo-A) and 1.3–2.0% (Apo-B), and the inter-assay CVs were 1.4–3.6% (Apo-A) and 2.7–2.9% (Apo-B). All the lipids were measured on a cobas 8000 modular analyzer (Roche Diagnostics, Penzberg, Germany). Cord blood betatrophin levels were determined using a commercially available ELISA kit (EIAab, Wuhan, China) according to the manufacturer’s instructions [20, 32]. Each sample was measured in triplicate.

### Measurement of mitochondria content

The mitochondria content was presented as the mitochondrial/nuclear DNA ratio (mtDNA/nDNA) and determined as previously described [33–35]. A fragment located in MT-ND1 (NC_012920.1) was amplified with the primers forward: TGGGCCATACGGTAGTATTTAGTTGG and reverse: TTACCCTATAGCACCCCCTCTAC for mitochondrial DNA; while a fragment in HBB (NG_000007.3) sequence were amplified with the primers forward: TTTTCCCACCCTTAGGCTG and reverse: CTCACTCAGTGTGGCAAAG for nuclear DNA [35]. The amplifications were performed in an ABI 7500 real-time PCR system (Life Technologies, Carlsbad, CA) with a 20 μl reaction mixture containing 80ng of purified DNA, 1 x SYBR Green/ROX qPCR Master Mix (Thermo Scientific, Waltham, MA) and 0.3 μM of each primer. The specificity of amplification was confirmed by melting curve analysis and agarose gel electrophoresis of the products. Each sample was measured in triplicate.

### Statistical analysis

A Kolmogorov-Smirnov analysis assessed the probability distributions of parameters except offspring parity and sex, which were analyzed by chi-square distribution. Normally distributed data are presented as mean ± SEM, and non-normally distributed data are presented as median (interquartile range). Comparisons between groups were compared by independent-sample t-test (normally distributed data) or Mann-Whitney U-test (non-normally distributed data). Differences in offspring parity and sex distribution were examined by chi-square test. Correlations between cord blood betatrophin and the normally distributed variables of interest (maternal gestational glycemic results, pre-pregnant BMI, gestational weight gain, cord blood HOMA-IR, TC, TG, LDL-C, Apo-A and Apo-B, placental mitochondrial content) were examined using Pearson’s correlation coefficients. Association between betatrophin and non-normally distributed variables including HDL were analysed by Spearman’s rank correlation coefficients. When appropriate, potential confounders (maternal gestational weight gain, pre-gestational BMI, age, offspring sex, birth weight, gestational age at delivery, parity, as well as cord blood HOMA-IR and lipid profile) were incorporated into the correlation analysis. The diagnostic value of betatrophin for GDM group was assessed using the area under the receiver operating characteristic (ROC) curve. A *P <*0.05 (two-tailed) was considered statistically significant. All statistical analyses were performed using SPSS software (version 13.0, Chicago, IL, USA).

## Results

Participants were divided into the GDM group and the Normoglycemia groups according to the IADPSG criteria [29, 30]. Eighty-two women (36 GDM and 46 Normoglycemia) with singleton pregnancy and underwent uncomplicated Cesarean delivery at our University Hospital were enrolled. Fifteen participants (6 GDM and 9 Normoglycemia) were excluded from the study since they did not met the study criteria abovementioned. Finally, after excluding 13 participants (7 GDM and 6 Normoglycemia) because of unavailable maternal gestational glucose records or cord blood samples, data from 54 enrollees (23 GDM and31 Normoglycemia) was eligible for analysis. Maternal and newborn descriptive information is presented in Table 1. The average glucose concentrations (fasting, 1h and 2h post-OGTT) at 24 to 28 weeks’ gestation and cord blood glucose levels were higher in GDM group (*P <* 0.05, Table 1). In addition, cord blood insulin also was higher in GDM trend (*P <* 0.1). Consistently, cord blood HOMA-IR was greater in the GDM group (*P <* 0.05, Table 1). Moreover, the cord blood TG also revealed a similar trend (*P <* 0.1, Table 1).

**Table 1:** Maternal and newborn characteristics.

	GDM	Normoglycemia
n	23	31
Mother's age (years)	32 (28, 34)	30 (26, 32)
Pre-pregnant weight (kg)	57.1±1.86	54.1±1.39
Pre-pregnant BMI (kg/m^2^)	21.6±0.59	20.5±0.47
Gestational weight gain (kg)	15.8±1.1	17.5±1.0
Fasting plasma glucose (mmol/L) at 24 to 28 weeks' gestation	5.0±0.09	4.3±0.08**
1-h post-OGTT glucose (mmol/L) at 24 to 28 weeks’ gestation	10.4±0.27	7.8±0.21**
2-h post-OGTT glucose (mmol/L) at 24 to 28 weeks’ gestation	8.7±0.30	6.5±0.15**
Cord blood insulin levels (mU/L)	6.7±0.60	5.1±0.43*
Cord blood glucose levels (mmol/L)	4.05±0.18	3.5±0.12*
Cord blood HOMA-IR	1.3±0.13	0.80±0.07*
Cord blood TC (mmol/l)	1.75±0.10	1.71±0.07
Cord blood TG (mmol/L)	0.20(0.12, 0.28)	0.15(0.11, 0.20)
Cord blood HDL-C (mmol/l)	0.75(0.61, 1.06)	0.71(0.58, 0.96)
Cord blood LDL-C (mmol/l)	0.55±0.04	0.58±0.03
Cord blood Apo-A (g/l)	0.87±0.04	0.80±0.02
Cord blood Apo-B (g/l)	0.18±0.01	0.18±0.01
Birth weight (kg)	3.3±0.10	3.2±0.06
Offspring sex (males/females)	9/14	13/18
Gestational age at delivery (days)	271.0±1.33	272.5±1.24
Parity (% primiparous)	774	78

Normally distributed data are presented as mean ± SEM, and non-normally distributed data are presented as median (interquartile range) except the offspring sex and parity are expressed as ratio.

* *P <* 0.05

** *P <* 0.01

**Fig 1:**
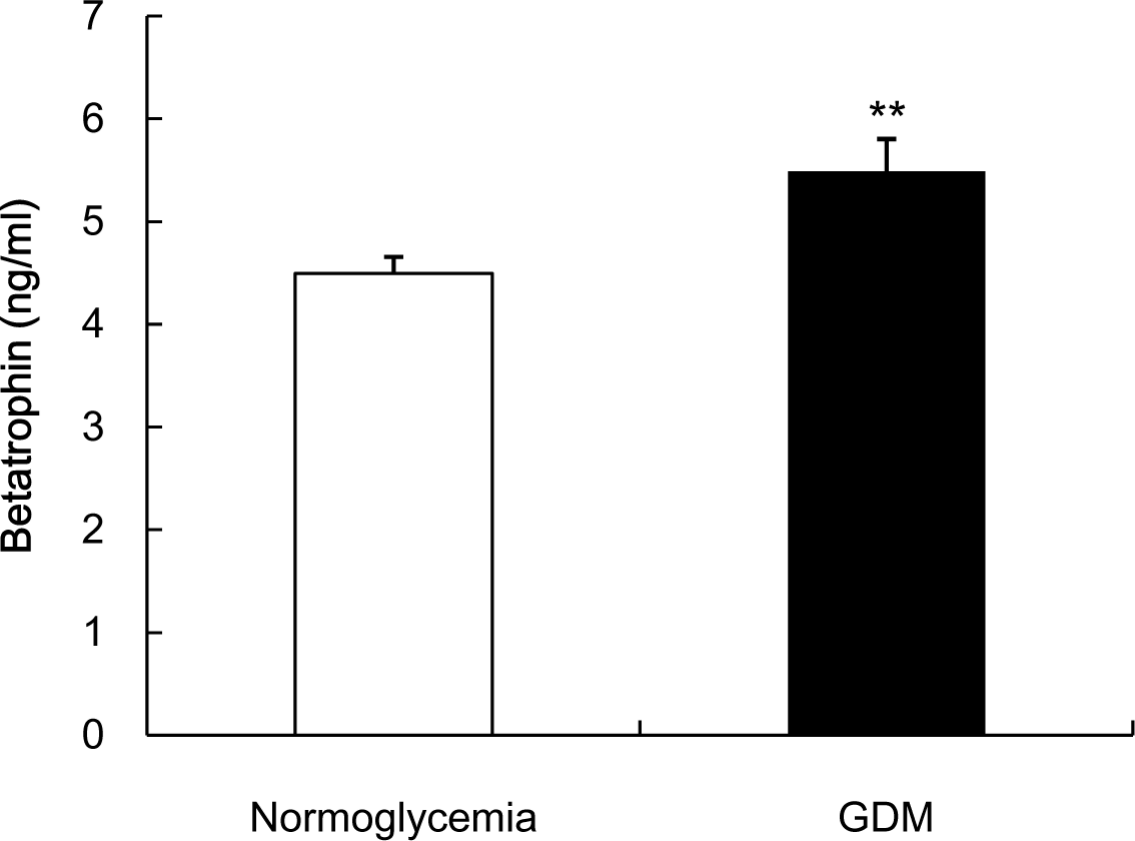
Cord blood betatropin is elevated in GDM group. Data is presented as mean ± SEM, **P < 0.01 compared with the normoglycemic group.

In humans, circulating betatrophin is elevated in type 2 diabetes [17–19], obesity [17], and type 1 diabetes [20]. Notably, in this study, cord blood betatrophin was significantly elevated in the GDM group compared with those who were euglycemic (*p*< 0.01, Fig 1). The ROC curve had an area under the curve (AUC) of 0.74, 95% confidence interval [CI] = 0.60 to 0.88, *p* = 0.003. Based on the ROC curve, a cutoff value of 5.3 ng/ml was chosen to predict the absence (cut-off value ≤5.3 ng/ml) or presence (cut-off value >5.3 ng/ml) of GDM (S1 Fig). Twenty-six of 35 (74.3%) normoglycemic mothers had a cord blood betatrophin values of 5.3 ng/ml or less; meanwhile, 14 of 19 (73.7%) GDM were identified by this cut-off value correctly, indicating that the positive predictive value was 0.737. Nine (39.1%) of the 23 GDM mothers had a cord blood betatrophin value less than 5.3 ng/ml, representing 60.9% sensitivity. The sensitivity and specificity were 0.609 and 0.839, respectively. Collectively, these data indicates that 40 of 54 (74.1%) could be diagnosed correctly as either with or without GDM by using this predictive model at cut-off value (5.3 ng/ml) (S1 Table).

**Fig 2:**
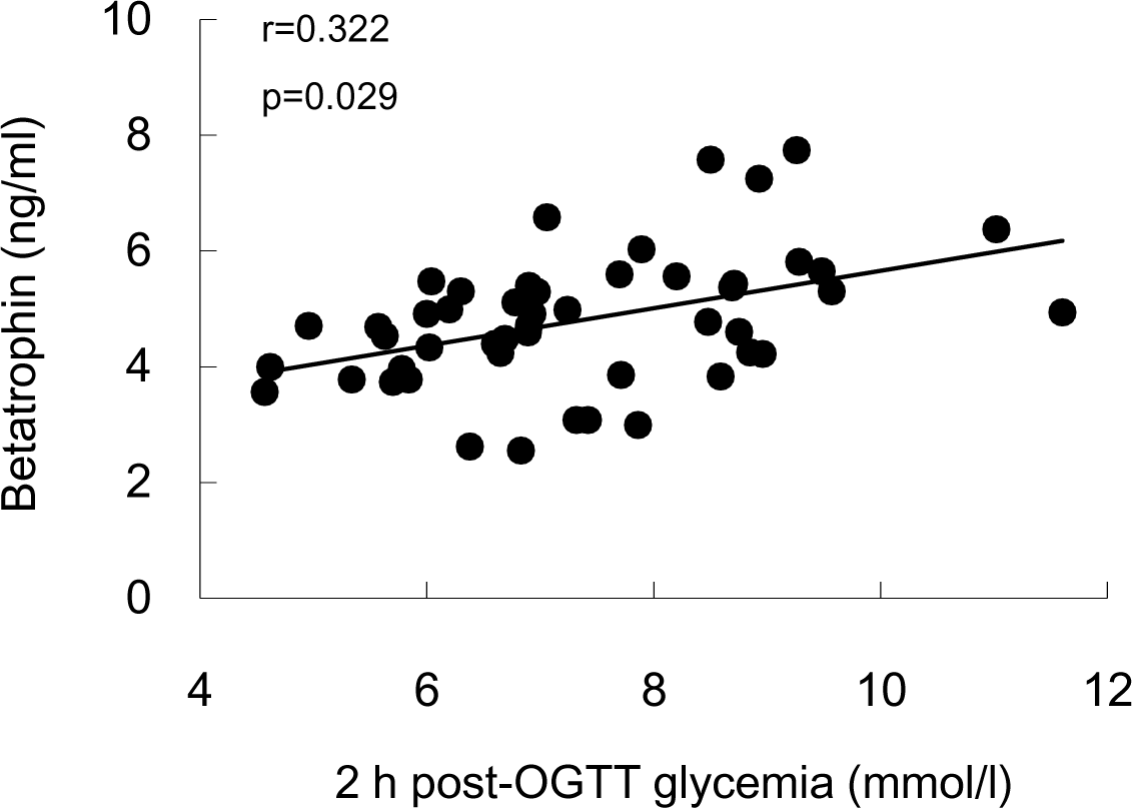
Correlation analysis between maternal gestational OGTT glycemia and cord blood betatrophin. Correlation was adjusted for pregestational BMI, gestational weight gain, cord blood HOMA-IR, newborn birth weight, sex, parity, and gestational age at delivery and maternal age. Data represents both cohorts (n = 54).

Cord blood betatrophin levels were also positively correlated with maternal gestational fasting, 1h and 2h post-OGTT glucose (*p*< 0.05), however, after adjusting for the potential confounders including cord blood HOMA-IR, lipid profile, maternal ages, pre-pregnant BMI, prenatal BMI, gestational weight gain, offspring parity, sex, birth weight and gestational age at delivery, only the 2h post-OGTT glycemia level significantly correlated with betatrophin level (Table 2, Fig 2).

**Table 2:** Pearson and Partial correlation coefficient between maternal gestational OGTT glucose concentration and cord blood betatrophin level in all participants.

		Fasting-OGTT glucose level (n = 54)		1h post-OGTT glucose levels (n = 54)		2h post-OGTT glucose level (n = 54)	
			*		*		*
Betatrophin	r_s_	**0.281**	0.273	**0.323**	**0.119**	**0.500**	**0.322**
	*p*	**0.039**	0.066	**0.017**	**0.429**	**0.000**	**0.029**

* Correlation were adjusted for pregestational BMI, gestational weight gain, cord blood HOMA-IR, newborn sex, birth weight, gestational age at delivery, parity and maternal age; correlations with *P <* 0.05 are in bold.

**Fig 3:**
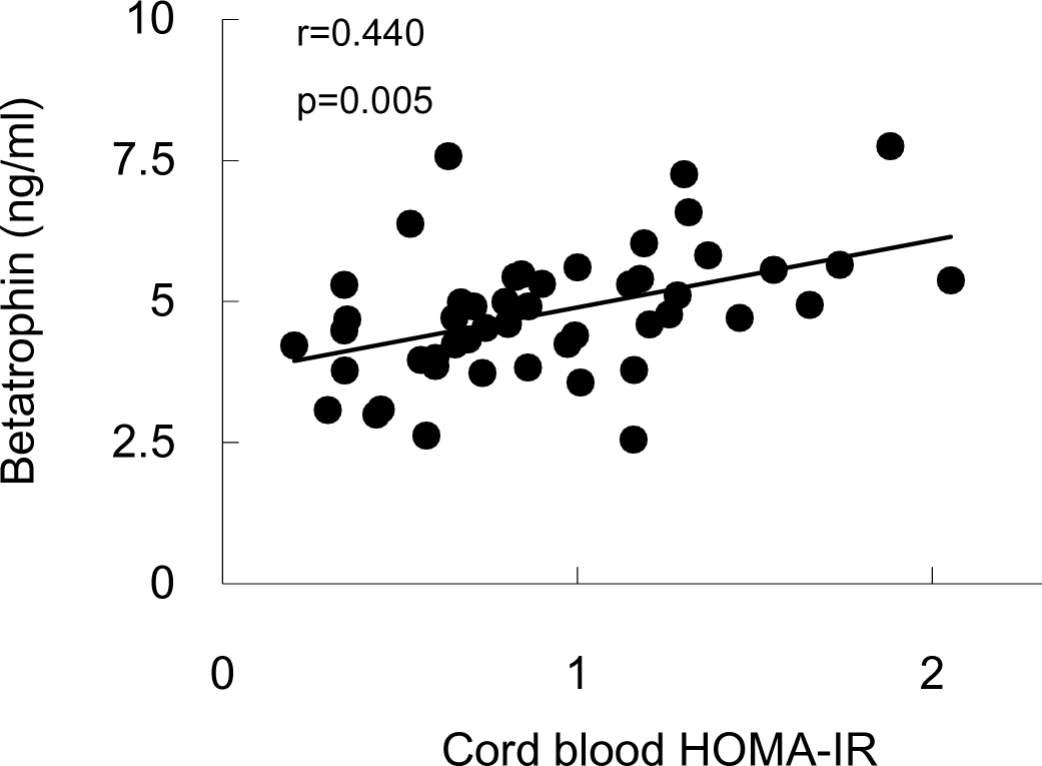
Correlation analysis between cord blood HOMA-IR and betatrophin. Correlation was adjusted for cord blood lipid profile, maternal gestational 2h post-OGTT glucose, ages, pre-pregnant BMI, prenatal BMI, gestational weight gain, offspring parity, sex, birth weight and gestational age at delivery. Data represents both cohorts (n = 54).

Betatrophin levels were significantly associated with insulin as well as HOMA-IR in cord blood (r = 0.556, *p* = 0.000 for insulin and r = 0.598, *p* = 0.000 for HOMA-IR), and the correlation remained significant with adjustment for the above-listed potential confounders (r = 0.457, *p* = 0.003 for insulin and r = 0.440, *p* = 0.005 for HOMA-IR, Fig 3).

**Fig 4:**
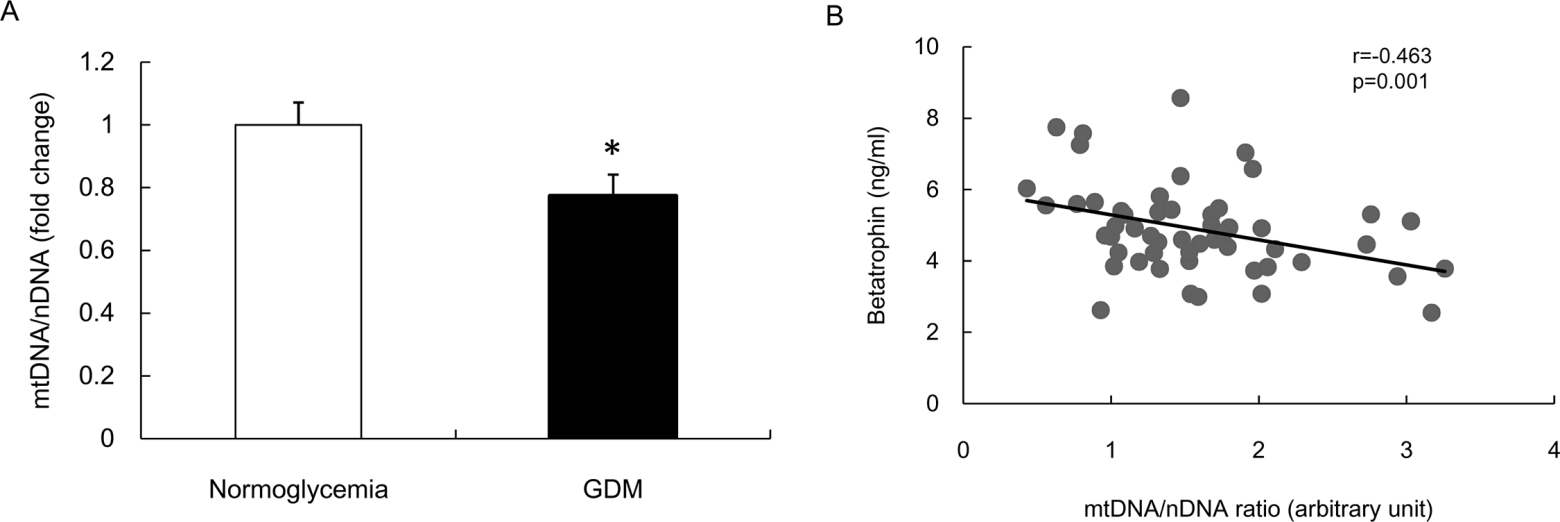
The placental mitochondrial content and the correlation analysis between cord blood betatrophin and placental mtDNA content. A. Reduced mitochondrial content (mtDNA/nDNA ratio) in GDM group. B. The correlation was adjusted for maternal age, pregestational BMI, gestational weight gain, cord blood HOMA-IR, newborn birth weight, sex, gestational age at delivery and parity.

The ratio of mtDNA per nucleus DNA in fetal placenta of GDM group was reduced (Fig 4A), indicative of attenuated mitochondrial content. Furthermore, cord blood betatrophin levels inversely correlated with the placental mtDNA/nDNA ratio, and the correlation was still significant after adjusting for the same potential confounders (r = -0.463, *p* = 0.001, Fig 4B).

Circulating betatrophin reportedly correlates with cholesterol and LDL-C levels [32], as well as HDL-C and triglyceride concentrations in human [36]. Interestingly, in this study, cord blood betatrophin was positively associated with Apo-A (r = 0.353, p = 0.009) and TG (r = 0.292, *p* = 0.032). No correlation was observed between betatrophin and LDL-C and HDL-C. However, after adjusting for potential confounders (as above), betatrophin did not significantly correlate with the lipid profiles except there remained a trend between betatrophin and Apo-A (r = 0.289, *p* = 0.051). In addition, cord blood betatrophin did not correlate with maternal pre-pregnant BMI or gestational weight gain (*p* > 0.05, data not shown).

## Discussion

Exposure of the fetus to intrauterine hyperglycemia may forebode the metabolic syndrome and diabetes in later life [6–8]. And, disturbingly, this may manifest early in childhood [11–13]. The secretion of betatrophin from liver, and possibly adipose, spurs pancreatic beta cell proliferation in response to insulin resistance, conceivably, in an attempt to restore glucose tolerance and mitigate lipid disturbances [15]. In concordance with this tenet, we found that cord blood betatrophin and HOMA-IR were significantly increased in the GDM group compared with the normoglycemia group. Moreover, cord betatrophin was positively correlated with maternal gestational glycemia and cord blood HOMA-IR, inferring that betatrophin could be a potential biomarker of intrauterine hyperglycemia and an indicator of insulin resistance in the offspring from GDM mothers. Admittedly, at this juncture, there is no proof that assessing betatrophin in cord blood at birth is a superior metric of fetal insult than serum glucose and insulin.

Numerous studies have found that exposure to intrauterine hyperglycemia can increase the future risk of obesity and type 2 diabetes early in life [11, 12, 14]. For example, a diabetic intrauterine milieu has been linked to adiposity and higher glucose and insulin levels at 5 years [11]. Additionally, in utero hyperglycemia may foretell an elevated systolic blood pressure juncture and HbA1c in childhood, posing a higher risk for cardiovascular disease and T2DM in later life [14]. A recent study found that maternal gestational glycemia inversely correlates with a child’s insulin sensitivity and beta cell response [12]—the implication being that intrauterine hyperglycemia programs for dysfunctional beta cell secretion as well as peripheral insulin target tissues [12]. GDM may predispose to fetal hyperinsulinemia [37] and, in our study, cord blood insulin did suggest such an association. Furthermore, in concert with other studies [38, 39], we found that the GDM newborn has reduced insulin sensitivity as evidenced by an increased cord blood HOMA-IR.

Betatrophin is expressed predominantly in adipose tissue and liver, and highly responsive to metabolic cues [15, 16]. Circulating betatrophin is increased with insulin resistance, such as type 2 diabetes [17–20] and obesity [17]. Interestingly, Fenzl et al. reported that betatrophin levels were similar between type 2 diabetic and non-diabetic cohort [32] while Gómez-Ambrosi et al. found that betatrophin was decreased in T2DM patients [40].

The placenta, serving as nutrient transporter, and gas exchanger for the fetus, plays a crucial role in fetal growth, development and future metabolic outcome [41]. The placenta is also recognized as an endocrine organ since it produces essential hormones for gestation. Insulin signaling in the placenta was reported to be impaired in pregnancies complicated by obesity and diabetes [42]. Moreover, placental function may be associated with pancreatic beta cell proliferation. For example, it can secret placental lactogen, which upregulates beta cell mass during gestation [43]. Conceivably, placenta may also produce betatrophin to regulate beta cell replication. In the current study, the cord betatrophin concentrations from GDM mothers were significantly increased, corroborating the very recent findings [44–47], and suggesting that intrauterine hyperglycemia may promote placental betatrophin secretion. Interestingly, a previous study found that placental betatrophin mRNA expression did not differ significantly between the GDM group and the control group [44].

Moreover, we observed that betatrophin levels were positively associated with maternal gestational glycemia. In humans, islet development and remodeling occur throughout the second trimester to early childhood [48]. Circulating betatrophin in humans is elevated 2 hours after a defined meal [17]. It is of note that only the 2h post-OGTT glycemia correlated with betatrophin levels, hence, this measurement may be a superior indicator of the fetal insulin resistance than the fasting or 1h post-OGTT glycemia. Moreover, cord blood betatrophin levels correlated positively with HOMA-IR, inferring an association between betatrophin and insulin sensitivity. Previous studies reported that betatrophin levels were increased significantly in mothers with GDM compared with the control group [44–46, 49]. Moreover, in the GDM group, there was a positive association between cord blood and maternal betatrophin levels [44]. Conceivably, the betatrophin levels were also elevated in the GDM mothers participating in this study, and correlated positively with the cord blood betatrophin levels.

Mitochondrial dysfunction is closely associated with impaired insulin sensitivity and diabetes [34, 50, 51], and mitochondrial deficiency contributes to mitochondrial dysfunction [34]. Similarly, mitochondria play a major role in placental function [52], and placental mitochondrial dysfunction may be a molecular mechanism which effectuates nutritional programming of insulin resistance [53]. We observed that the mtDNA/nDNA ratio was decreased in placenta of GDM group, indicative of reduced mitochondrial content and impaired mitochondrial function. Moreover, the mtDNA/nDNA ratio inversely correlated with cord blood betatrophin levels. Although we found a correlation between betatrophin and placental mitochondrial content, there is no evidence that the alteration in circulating betatrophin levels leads to the change in mitochondrial content, both of which are likely a resultant of intrauterine hyperglycemia.

Dyslipidemia is a common morbidity associated with GDM and type 2 diabetes. Given the reports that betatrophin correlates positively with serum TG, we found a similar trend in the cord blood, namely, increased concentrations of both TG and betatrophin in the GDM cohort.

Reported betatrophin levels in obesity are inconsistent. Some reports found that betatrophin was increased [17], whereas in other studies it was reduced [40] or unaltered [32]. We found no correlation between betatrophin and maternal pre-pregnant BMI or gestational weight gain.

All statistical correlations analyzed herein have been adjusted for potential confounders to avoid misleading conclusions. However, some limitations must be acknowledged. First, since the current study was carried out in a homogeneous population of Chinese Han ethnicity, the findings may not be generalizable to other populations. Besides, the sample size was relatively small which may impede the detection of weak correlations between betatrophin levels and some metabolic parameters such as lipid profile. Although we observed positive correlations between fetal betatrophin and maternal gestational glycemia, as well as fetal cord HOMA-IR, we cannot conclude that maternal gestational hyperglycemia begets fetal betatrophin hypersecretion given the limitations of a cross- sectional study design. Additionally, the gestational glycemia data from a sole OGTT at 24–28 week of gestation may not correctly represent the glycemic status throughout the entire gestation. Also, the long term maternal glucose control and the child's outcomes are unknown and here we can not make cause and effect incontrovertible conclusions based on a cross sectional study design and correlation analysis. A future prospective study for confirmation is warranted.

In conclusion, we found that cord blood betatrophin was increased in GDM mothers at delivery, and betatrophin concentrations correlated significantly with HOMA-IR and maternal gestational glycemia. The inference is that cord blood betatrophin may be a potential biomarker of maternal intrauterine hyperglycemia and fetal insulin resistance which, ultimately, may portend later metabolic disturbances, even as early in childhood.

## Supporting Information

S1 Fig The diagnostic value of betatrophin for GDM was assessed by an ROC curve, with an area under the curve (AUC) of 0.74, 95% confidence interval [CI] = 0.60 to 0.88, P = 0.003. Cut-off value (5.3 ng/ml) is depicted.
(TIF)

S1 Table The diagnostic value of betatrophin for GDM and normoglycemia mothers. (DOCX)

## References

[1] Barker DJ, Hales CN, Fall CH, Osmond C, Phipps K, Clark PM. Type 2 (non-insulin-dependent) diabetes mellitus, hypertension and hyperlipidaemia (syndrome X): relation to reduced fetal growth. Diabetologia. 1993;36(1):62–7. Epub 1993/01/01. . 8436255 10.1007/BF00399095

[2] Hales CN, Barker DJ. Type 2 (non-insulin-dependent) diabetes mellitus: the thrifty phenotype hypothesis. Diabetologia. 1992;35(7):595–601. Epub 1992/07/01. . 1644236 10.1007/BF00400248

[3] Sarr O, Yang K, Regnault TR. In utero programming of later adiposity: the role of fetal growth restriction. Journal of pregnancy. 2012;2012:134758 Epub 2012/12/20. 10.1155/2012/134758 23251802 PMC3518064

[4] Jiwani A, Marseille E, Lohse N, Damm P, Hod M, Kahn JG. Gestational diabetes mellitus: results from a survey of country prevalence and practices. The journal of maternal-fetal & neonatal medicine: the official journal of the European Association of Perinatal Medicine, the Federation of Asia and Oceania Perinatal Societies, the International Society of Perinatal Obstet. 2012;25(6):600–10. Epub 2011/07/19. 10.3109/14767058.2011.587921 . 21762003

[5] Norman JE, Reynolds RM. The consequences of obesity and excess weight gain in pregnancy. The Proceedings of the Nutrition Society. 2011;70(4):450–6. Epub 2011/09/02. 10.1017/s0029665111003077 . 21880162

[6] Clausen TD, Mathiesen ER, Hansen T, Pedersen O, Jensen DM, Lauenborg J, et al Overweight and the metabolic syndrome in adult offspring of women with diet-treated gestational diabetes mellitus or type 1 diabetes. The Journal of clinical endocrinology and metabolism. 2009;94(7):2464–70. Epub 2009/05/07. 10.1210/jc.2009-0305 . 19417040

[7] Damm P. Future risk of diabetes in mother and child after gestational diabetes mellitus. International journal of gynaecology and obstetrics: the official organ of the International Federation of Gynaecology and Obstetrics. 2009;104 Suppl 1:S25–6. Epub 2009/01/20. 10.1016/j.ijgo.2008.11.025 . 19150058

[8] Lawlor DA, Lichtenstein P, Langstrom N. Association of maternal diabetes mellitus in pregnancy with offspring adiposity into early adulthood: sibling study in a prospective cohort of 280,866 men from 248,293 families. Circulation. 2011;123(3):258–65. Epub 2011/01/12. 10.1161/circulationaha.110.980169 21220735 PMC4440894

[9] Zinkhan EK, Fu Q, Wang Y, Yu X, Callaway CW, Segar JL, et al Maternal Hyperglycemia Disrupts Histone 3 Lysine 36 Trimethylation of the IGF-1 Gene. Journal of nutrition and metabolism. 2012;2012:930364 Epub 2012/05/02. 10.1155/2012/930364 22548154 PMC3324902

[10] Kousta E, Lawrence NJ, Godsland IF, Penny A, Anyaoku V, Millauer BA, et al Insulin resistance and beta-cell dysfunction in normoglycaemic European women with a history of gestational diabetes. Clinical endocrinology. 2003;59(3):289–97. Epub 2003/08/16. . 12919151 10.1046/j.1365-2265.2003.01820.x

[11] Krishnaveni GV, Hill JC, Leary SD, Veena SR, Saperia J, Saroja A, et al Anthropometry, glucose tolerance, and insulin concentrations in Indian children: relationships to maternal glucose and insulin concentrations during pregnancy. Diabetes care. 2005;28(12):2919–25. Epub 2005/11/25. . 16306555 10.2337/diacare.28.12.2919

[12] Bush NC, Chandler-Laney PC, Rouse DJ, Granger WM, Oster RA, Gower BA. Higher maternal gestational glucose concentration is associated with lower offspring insulin sensitivity and altered beta-cell function. The Journal of clinical endocrinology and metabolism. 2011;96(5):E803–9. Epub 2011/02/25. 10.1210/jc.2010-2902 21346075 PMC3176781

[13] Page KA, Romero A, Buchanan TA, Xiang AH. Gestational diabetes mellitus, maternal obesity, and adiposity in offspring. The Journal of pediatrics. 2014;164(4):807–10. Epub 2014/01/07. 10.1016/j.jpeds.2013.11.063 24388326 PMC3962700

[14] Bunt JC, Tataranni PA, Salbe AD. Intrauterine exposure to diabetes is a determinant of hemoglobin A(1)c and systolic blood pressure in pima Indian children. The Journal of clinical endocrinology and metabolism. 2005;90(6):3225–9. Epub 2005/03/31. 10.1210/jc.2005-0007 15797952 PMC1579248

[15] Zhu JZ, Yu CH, Li YM. Betatrophin provides a new insight into diabetes treatment and lipid metabolism (Review). Biomedical reports. 2014;2(4):447–51. Epub 2014/06/20. 10.3892/br.2014.284 24944788 PMC4051489

[16] Zhang R, Abou-Samra AB. A dual role of lipasin (betatrophin) in lipid metabolism and glucose homeostasis: consensus and controversy. Cardiovascular diabetology. 2014;13(1):133 Epub 2014/09/13. 10.1186/preaccept-6310273751364807 25212743 PMC4172915

[17] Fu Z, Berhane F, Fite A, Seyoum B, Abou-Samra AB, Zhang R. Elevated circulating lipasin/betatrophin in human type 2 diabetes and obesity. Scientific reports. 2014;4:5013 Epub 2014/05/24. 10.1038/srep05013 . 24852694 PMC5381405

[18] Espes D, Martinell M, Carlsson PO. Increased circulating betatrophin concentrations in patients with type 2 diabetes. International journal of endocrinology. 2014;2014:323407 Epub 2014/06/26. 10.1155/2014/323407 24963292 PMC4055101

[19] Hu H, Sun W, Yu S, Hong X, Qian W, Tang B, et al Increased circulating levels of betatrophin in newly diagnosed type 2 diabetic patients. Diabetes care. 2014;37(10):2718–22. Epub 2014/07/16. 10.2337/dc14-0602 . 25024395

[20] Espes D, Lau J, Carlsson PO. Increased circulating levels of betatrophin in individuals with long-standing type 1 diabetes. Diabetologia. 2014;57(1):50–3. Epub 2013/10/01. 10.1007/s00125-013-3071-1 24078058 PMC3855541

[21] Rieck S, White P, Schug J, Fox AJ, Smirnova O, Gao N, et al The transcriptional response of the islet to pregnancy in mice. Molecular endocrinology (Baltimore, Md). 2009;23(10):1702–12. Epub 2009/07/04. 10.1210/me.2009-0144 19574445 PMC2754894

[22] Alonso LC, Yokoe T, Zhang P, Scott DK, Kim SK, O'Donnell CP, et al Glucose infusion in mice: a new model to induce beta-cell replication. Diabetes. 2007;56(7):1792–801. Epub 2007/04/03. 10.2337/db06-1513 17400928 PMC2921922

[23] Yi P, Park JS, Melton DA. Betatrophin: a hormone that controls pancreatic beta cell proliferation. Cell. 2013;153(4):747–58. Epub 2013/04/30. 10.1016/j.cell.2013.04.008 23623304 PMC3756510

[24] Gusarova V, Alexa CA, Na E, Stevis PE, Xin Y, Bonner-Weir S, et al ANGPTL8/betatrophin does not control pancreatic beta cell expansion. Cell. 2014;159(3):691–6. Epub 2014/11/25. 10.1016/j.cell.2014.09.027 25417115 PMC4243040

[25] Wang Y, Quagliarini F, Gusarova V, Gromada J, Valenzuela DM, Cohen JC, et al Mice lacking ANGPTL8 (Betatrophin) manifest disrupted triglyceride metabolism without impaired glucose homeostasis. Proceedings of the National Academy of Sciences of the United States of America. 2013;110(40):16109–14. Epub 2013/09/18. 10.1073/pnas.1315292110 24043787 PMC3791734

[26] Zhang R. Lipasin, a novel nutritionally-regulated liver-enriched factor that regulates serum triglyceride levels. Biochemical and biophysical research communications. 2012;424(4):786–92. Epub 2012/07/20. 10.1016/j.bbrc.2012.07.038 . 22809513

[27] Mooradian AD. Dyslipidemia in type 2 diabetes mellitus. Nature clinical practice Endocrinology & metabolism. 2009;5(3):150–9. Epub 2009/02/21. 10.1038/ncpendmet1066 . 19229235

[28] Li G, Kong L, Zhang L, Fan L, Su Y, Rose JC, et al Early Pregnancy Maternal Lipid Profiles and the Risk of Gestational Diabetes Mellitus Stratified for Body Mass Index. Reproductive sciences (Thousand Oaks, Calif). 2014 Epub 2014/11/15. 10.1177/1933719114557896 . 25394643 PMC4502803

[29] International Association of D, Pregnancy Study Groups Consensus P, Metzger BE, Gabbe SG, Persson B, Buchanan TA, et al International association of diabetes and pregnancy study groups recommendations on the diagnosis and classification of hyperglycemia in pregnancy. Diabetes care. 2010;33(3):676–82. Epub 2010/03/02. 10.2337/dc09-1848 20190296 PMC2827530

[30] American Diabetes A. Standards of medical care in diabetes—2014. Diabetes care. 2014;37 Suppl 1:S14–80. Epub 2013/12/21. 10.2337/dc14-S014 . 24357209

[31] Xie X, Gao H, Zeng W, Chen S, Feng L, Deng D, et al Placental DNA methylation of peroxisome-proliferator-activated receptor-gamma co-activator-1alpha promoter is associated with maternal gestational glucose level. Clinical science (London, England: 1979). 2015;129(4):385–94. Epub 2015/04/16. 10.1042/cs20140688 . 25875376

[32] Fenzl A, Itariu BK, Kosi L, Fritzer-Szekeres M, Kautzky-Willer A, Stulnig TM, et al Circulating betatrophin correlates with atherogenic lipid profiles but not with glucose and insulin levels in insulin-resistant individuals. Diabetologia. 2014;57(6):1204–8. Epub 2014/03/14. 10.1007/s00125-014-3208-x . 24623100

[33] Xie X, Lin T, Zhang M, Liao L, Yuan G, Gao H, et al IUGR with infantile overnutrition programs an insulin-resistant phenotype through DNA methylation of peroxisome proliferator-activated receptor-gamma coactivator-1alpha in rats. Pediatric research. 2015;77(5):625–32. Epub 2015/02/13. 10.1038/pr.2015.32 . 25675425

[34] Barres R, Osler ME, Yan J, Rune A, Fritz T, Caidahl K, et al Non-CpG methylation of the PGC-1alpha promoter through DNMT3B controls mitochondrial density. Cell metabolism. 2009;10(3):189–98. Epub 2009/09/03. 10.1016/j.cmet.2009.07.011 . 19723495

[35] Reiling E, Ling C, Uitterlinden AG, Van't Riet E, Welschen LM, Ladenvall C, et al The association of mitochondrial content with prevalent and incident type 2 diabetes. The Journal of clinical endocrinology and metabolism. 2010;95(4):1909–15. Epub 2010/02/13. 10.1210/jc.2009-1775 . 20150578

[36] Gomez-Ambrosi J, Pascual E, Catalan V, Rodriguez A, Ramirez B, Silva C, et al Circulating betatrophin concentrations are decreased in human obesity and type 2 diabetes. The Journal of clinical endocrinology and metabolism. 2014;99(10):E2004–9. Epub 2014/07/23. 10.1210/jc.2014-1568 . 25050901

[37] Westgate JA, Lindsay RS, Beattie J, Pattison NS, Gamble G, Mildenhall LF, et al Hyperinsulinemia in cord blood in mothers with type 2 diabetes and gestational diabetes mellitus in New Zealand. Diabetes care. 2006;29(6):1345–50. Epub 2006/05/30. 10.2337/dc05-1677 . 16732019

[38] Luo ZC, Nuyt AM, Delvin E, Fraser WD, Julien P, Audibert F, et al Maternal and fetal leptin, adiponectin levels and associations with fetal insulin sensitivity. Obesity (Silver Spring, Md). 2013;21(1):210–6. Epub 2013/03/19. 10.1002/oby.20250 . 23505188

[39] Wang Q, Huang R, Yu B, Cao F, Wang H, Zhang M, et al Higher fetal insulin resistance in Chinese pregnant women with gestational diabetes mellitus and correlation with maternal insulin resistance. PloS one. 2013;8(4):e59845 Epub 2013/04/06. 10.1371/journal.pone.0059845 23560057 PMC3613391

[40] Gomez-Ambrosi J, Pascual E, Catalan V, Rodriguez A, Ramirez B, Silva C, et al Circulating Betatrophin Concentrations Are Decreased in Human Obesity and Type 2 Diabetes. The Journal of clinical endocrinology and metabolism. 2014 10.1210/jc.2014-1568 . 25050901

[41] Nelissen EC, van Montfoort AP, Dumoulin JC, Evers JL. Epigenetics and the placenta. Human reproduction update. 2011;17(3):397–417. Epub 2010/10/21. 10.1093/humupd/dmq052 . 20959349

[42] Colomiere M, Permezel M, Riley C, Desoye G, Lappas M. Defective insulin signaling in placenta from pregnancies complicated by gestational diabetes mellitus. European journal of endocrinology / European Federation of Endocrine Societies. 2009;160(4):567–78. Epub 2009/01/31. 10.1530/eje-09-0031 . 19179458

[43] Sorenson RL, Brelje TC. Adaptation of islets of Langerhans to pregnancy: beta-cell growth, enhanced insulin secretion and the role of lactogenic hormones. Hormone and metabolic research = Hormon- und Stoffwechselforschung = Hormones et metabolisme. 1997;29(6):301–7. Epub 1997/06/01. 10.1055/s-2007-979040 . 9230352

[44] Wawrusiewicz-Kurylonek N, Telejko B, Kuzmicki M, Sobota A, Lipinska D, Pliszka J, et al Increased Maternal and Cord Blood Betatrophin in Gestational Diabetes. PloS one. 2015;10(6):e0131171 Epub 2015/06/27. 10.1371/journal.pone.0131171 26115519 PMC4483159

[45] Trebotic LK, Klimek P, Thomas A, Fenzl A, Leitner K, Springer S, et al Circulating Betatrophin Is Strongly Increased in Pregnancy and Gestational Diabetes Mellitus. PloS one. 2015;10(9):e0136701 Epub 2015/09/02. 10.1371/journal.pone.0136701 26325425 PMC4556632

[46] Ebert T, Kralisch S, Wurst U, Lossner U, Kratzsch J, Bluher M, et al Betatrophin levels are increased in women with gestational diabetes mellitus compared to healthy pregnant controls. European journal of endocrinology / European Federation of Endocrine Societies. 2015;173(1):1–7. Epub 2015/04/09. 10.1530/eje-14-0815 . 25850828

[47] Erol O, Ellidag HY, Ayik H, Ozel MK, Derbent AU, Yilmaz N. Evaluation of circulating betatrophin levels in gestational diabetes mellitus. Gynecological endocrinology: the official journal of the International Society of Gynecological Endocrinology. 2015:1–5. Epub 2015/08/06. . 26291796 10.3109/09513590.2015.1056142

[48] Fowden AL, Hill DJ. Intra-uterine programming of the endocrine pancreas. British medical bulletin. 2001;60:123–42. Epub 2002/01/26. . 11809622 10.1093/bmb/60.1.123

[49] Erol O, Ellidag HY, Ayik H, Ozel MK, Derbent AU, Yilmaz N. Evaluation of circulating betatrophin levels in gestational diabetes mellitus. Gynecological endocrinology: the official journal of the International Society of Gynecological Endocrinology. 2015;31(8):652–6. Epub 2015/08/21. 10.3109/09513590.2015.1056142 . 26291796

[50] Handschin C, Spiegelman BM. Peroxisome proliferator-activated receptor gamma coactivator 1 coactivators, energy homeostasis, and metabolism. Endocr Rev. 2006;27(7):728–35. Epub 2006/10/05. 10.1210/er.2006-0037 . 17018837

[51] Newsholme P, Haber EP, Hirabara SM, Rebelato EL, Procopio J, Morgan D, et al Diabetes associated cell stress and dysfunction: role of mitochondrial and non-mitochondrial ROS production and activity. The Journal of physiology. 2007;583(Pt 1):9–24. Epub 2007/06/23. 10.1113/jphysiol.2007.135871 17584843 PMC2277225

[52] Chiaratti MR, Malik S, Diot A, Rapa E, Macleod L, Morten K, et al Is Placental Mitochondrial Function a Regulator that Matches Fetal and Placental Growth to Maternal Nutrient Intake in the Mouse? PloS one. 2015;10(7):e0130631 Epub 2015/07/02. 10.1371/journal.pone.0130631 26132581 PMC4488591

[53] Duque-Guimaraes DE, Ozanne SE. Nutritional programming of insulin resistance: causes and consequences. Trends in endocrinology and metabolism: TEM. 2013;24(10):525–35. Epub 2013/06/25. 10.1016/j.tem.2013.05.006 . 23791137

