# Supplementary figures and images for: Increased Cord Blood Betatrophin Levels in the Offspring of Mothers with Gestational Diabetes
Source: PLoS One. 2016 May 19;11(5):e0155646. doi: 10.1371/journal.pone.0155646 (PMC4873017; doi:10.1371/journal.pone.0155646)

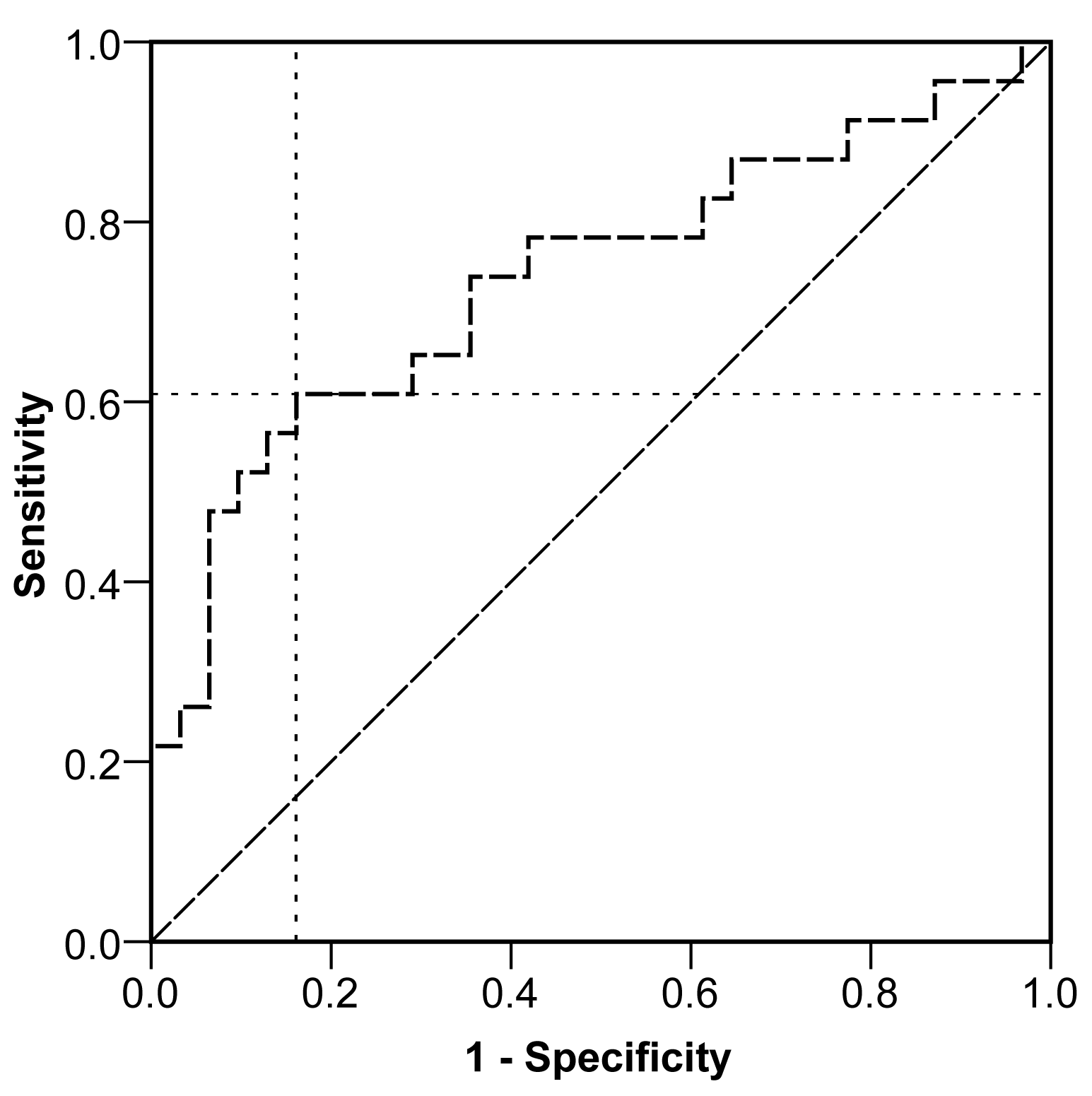

Supplement: S1 Fig — Cut-off value (5.3 ng/ml) is depicted. (TIF) [file pone.0155646.s001.tif]
