# Supplementary material for: Increased Cord Blood Betatrophin Levels in the Offspring of Mothers with Gestational Diabetes
Source: PLoS One. 2016 May 19;11(5):e0155646. doi: 10.1371/journal.pone.0155646 (PMC4873017; doi:10.1371/journal.pone.0155646)
Supplement: S1 Table — (DOCX) [file pone.0155646.s002.docx]

**S1 Table.** The diagnostic value of betatrophin for GDM and normoglycemia mothers

| GDM predicted  by betatrophin level | All participants | Clinical diagnosis | |
| --- | --- | --- | --- |
|  |  | Normoglycemia | GDM |
| Cut-off value (5.3 ng/ml)  Normoglycemia (≤5.3 ng/ml)  GDM (>5.3 ng/ml) | 35 (64.8%)  19 (35.2%) | 26 (83.9%)  5 (16.1%) | 9 (39.1%)  14 (60.9%) |
